# Stem cell therapy for regenerating periodontal bony defects: A narrative review

**DOI:** 10.34172/japid.025.3749

**Published:** 2025-03-03

**Authors:** Samira Mohammad Mirzapour, Fatemeh Jalali

**Affiliations:** ^1^Department of Periodontics, Faculty of Dentistry, Tabriz University of Medical Sciences, Tabriz, Iran; ^2^Student Research Committee, Faculty of Dentistry, Tabriz University of Medical Sciences, Tabriz, Iran

**Keywords:** Periodontitis, Regeneration, Review, Stem cells

## Abstract

Periodontal bony defects pose a significant challenge in periodontology, necessitating advanced regenerative approaches to restore the lost structures. Stem cell-based therapies have emerged as a promising solution due to their ability to differentiate into various cells, modulating the regenerative microenvironment. This narrative review explores the potential of stem cells derived from multiple sources in treating periodontal bony defects. Additionally, we examine evidence from both animal and human studies, highlighting advancements, clinical outcomes, and limitations. By investigating these findings, this article provides a comprehensive overview of the advantages of stem cell-based therapies compared to other regenerative techniques in addressing periodontal bony defects and discusses the limitations of their translation into routine clinical practice.

## Introduction

 Periodontitis represents a persistent, multifaceted condition that impacts the soft and hard tissues supporting the dentition, influencing over 50% of the adult population. Periodontal defects are caused by inflammation and related destruction of the periodontium. The periodontium is the tooth-supporting complex containing the gingiva, alveolar bone, periodontal ligament (PDL), and cementum.^1-3^ Most teeth are extracted due to periodontal disease. Periodontitis is also related to systemic diseases. Periodontal pathogenic factors are critical risk elements for neurodegenerative conditions (specifically Alzheimer’s disease), diabetic conditions, cardiac disorders, malignancies, immune-related disorders, and a variety of other systemic diseases.^4,5^ Therefore, it seems that the treatment of periodontitis is critical to improving overall health.

 Conventional therapies for periodontitis include improving oral hygiene, scaling and root planing, and periodontal surgery to access the subgingival areas. These treatment methods are usually effective for preventing disease progression but cannot regenerate lost tissues. Therefore, finding new therapeutic approaches that can regenerate periodontal tissues is necessary.

 Various periodontal regenerative strategies exist, including guided tissue regeneration (GTR), induced tissue regeneration (ITR), and stem cell therapy. The basis of GTR is to use a membrane that excludes the supra-crestal cells and leads to the proliferation of periodontium. ITR is based on applying enamel matrix derivatives (EMDs), which attract the progenitor cells to the area, modulate inflammation, and promote angiogenesis.^1,2,4,6-8^ The third option is based on stem cells, primitive cell groups capable of maturing into specialized cell forms.

 Three types of stem cells can be distinguished: somatic stem cells, induced pluripotent stem cells (iPSCs), and embryonic stem cells (ESCs). Hematopoietic, mesenchymal, intestinal, neuronal, epidermal, and hair follicle stem cells are among the varieties of somatic stem cells.^9^ Somatic stem cells, particularly mesenchymal stem cells (MSCs), are frequently used due to ethical issues regarding ESCs and the unpredictable nature of iPSCs.^10^

**Figure 1:**
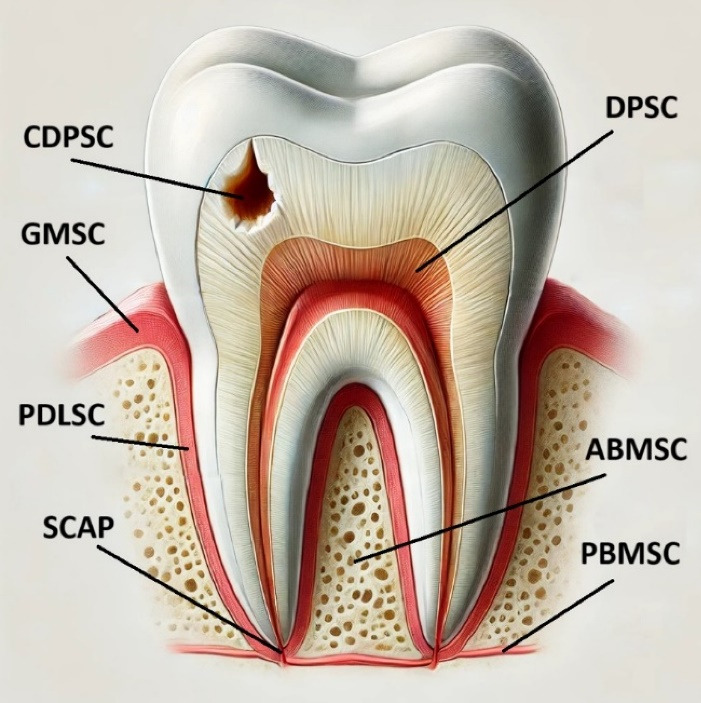
The most common odontogenic stem cell resources. These include the DPSC (dental pulp stem cells), ABMSC (alveolar bone marrow stem cells), PBMSC (peripheral blood mesenchymal stem cells), PDLSC (periodontal ligament stem cells), GMSC (gingival mesenchymal stem cells), SCAP (stem cells from the apical papilla), and CDPSC (carious dental pulp stem cells). The image was created with the help of ChatGPT-4

 MSCs are capable of improving periodontal tissue regeneration. This regenerative potential means they could be a significant factor in reconstructing tissues lost due to periodontitis. MSCs have different resources, including blood from the umbilical cord, bone marrow, adipose tissue, skeletal muscles, etc. Also, MSCs can be gained from intraoral tissues such as dental pulp, dental follicles, and gingival connective tissues.^11,12^Figure 1 summarizes different sources of stem cells. More research on using stem cells to regenerate periodontal tissues has recently been published, and the field is still developing. This study aimed to determine whether stem cell treatment could effectively treat periodontal bone abnormalities.

## Periodontal bony defects

 Various classifications are used for bony defects.^13^ According to Goldman and Cohen’s classification, periodontal defects can be divided into “infra-bony” and “supra-bony.” The base of the periodontal pocket is located coronally to the alveolar crest in supra-bony defects and apically in infra-bony defects. In another classification, Papapanou and Tonetti divided bony defects into supra-bony, infra-bony, and inter-radicular or furcation.^14^ The efficacy of stem cell application is closely associated with the morphology of the defect.^13,15^ Periodontal bony defects vary in morphology and regenerative potential, primarily based on whether the defect is contained or non-contained. The configuration of the defect directly affects the ability to achieve successful regeneration. Contained defects, also known as infra-bony defects, have bony walls surrounding the defect, creating a “contained” environment such as three-wall defects. These walls support the graft material and provide a favorable architecture for regeneration. The surrounding bone enhances cell migration, vascularization, and wound stability.^14^

 Retaining the graft material and promoting regeneration are challenging in non-contained defects since they lack sufficient surrounding bone walls. The absence of walls requires additional efforts to create a regenerative-friendly environment. Advanced techniques like 3D-printed scaffolds with stem cell therapy may improve outcomes. Stem cells can differentiate into osteoblasts, fibroblasts (PDL cells), and cementoblasts. This capability directly facilitates the formation of the triad of periodontal tissues required for regeneration. These cells secrete bioactive molecules like growth factors and cytokines, stimulating angiogenesis, modulating inflammation, and promoting regeneration. Injectable hydrogels or microspheres loaded with stem cells can fill irregularly shaped non-contained defects, adapting to the defect’s morphology. Though challenges remain, ongoing advancements in biomaterials and regenerative medicine hold great promise for the future of periodontal therapy.^16,17^

## Key factors for regeneration

**Figure 2:**
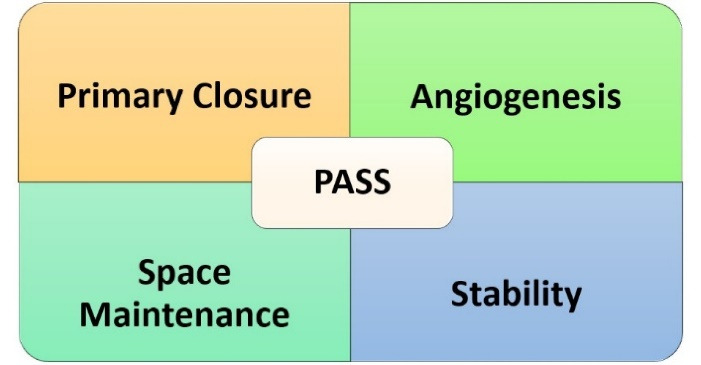
The four key biological principles, PASS (primary wound closure, angiogenesis, space maintenance, and stability of the wound), which are crucial for predictable bone regeneration

 Wang et al^18^ outlined the four fundamental biological principles, collectively known as PASS, which are essential for predictable bone regeneration. As illustrated in Figure 2, these principles include ensuring the primary closure of the wound, facilitating angiogenesis to prepare the crucial vascular supply, maintaining or creating adequate space, and ensuring the stability of the wound and the blood clot to promote regeneration.

 Primary closure of the soft tissue flap over the defect is critical to protect the regenerative area and prevent bacterial contamination. Stem cells thrive in a stable, isolated environment. Stem cells rely on a well-vascularized environment for survival and differentiation. Therefore, angiogenesis is essential for creating new bone, and dental stem cells are directly involved in the process by transforming into endothelial cells. Additionally, they encourage the development of vessels by releasing paracrine angiogenesis-inducing factors.^19,20^ Human leukocyte antigen G5, transforming growth factor, prostaglandin E2 and indolamine 2,3-dioxygenase are among the crucial elements that promote stem cell regeneration.^21-24^

 A stable three-dimensional scaffold or structure is required to maintain the defect space for tissue growth and prevent soft tissue collapse into the defect. Stem cells are often loaded on biodegradable scaffolds (e.g., collagen, hydroxyapatite, or bioactive ceramics) that maintain the defect’s architecture. Distinct platforms are being created and tried to encourage the extraordinary bone-forming potential of dental mesenchymal stem cells (DMSCs). Thus, cells that can promote intrinsic differentiation must exist for stem cell-based treatments.^25,26^

 Mechanical stability of the wound site is crucial to avoid disrupting the regenerative process and allow proper integration of new tissues. Micromovements can disrupt the attachment and differentiation of stem cells, impeding bone and PDL regeneration. The donor contexts and receptors are crucial in assessing the regenerative capability of transplanted MSCs during stem cell treatments.^27^ A sufficient supply of MSCs with osteogenic differentiation potential, appropriate bioactive substances to guide this differentiation, and scaffold biomaterials that support cellular interactions are three essential components of bone tissue engineering using stem cells. As a crucial factor, the availability of MSC promotes osteoblast formation and bone regeneration, whereas bioactive materials produce a favorable environment. However, scaffold biomaterials provide the structural framework required for the attachment of cells, differentiation, and proliferation. These components work together for effective bone regeneration.^28-30^

## Types of stem cells

 As presented in Table 1, stem cells can be categorized into several types, including embryonic, induced pluripotent, and various subgroups of somatic stem cells, which are further discussed below.

**Table 1:** Different types of stem cells

**Types of stem cells**				
Embryonic stem cells (ESCs)	Induced pluripotent stem cells (iPSCs)	Somatic stem cells group
		Dental pulp stem cells (DPSC)
		Carious Dental Pulp Stem Cells (CDPSC)
		Stem cells from human exfoliated deciduous teeth (SHED)
		Periodontal ligament stem cells (PDLSC)
		Dental follicle stem cells (DFSC)
		Stem cells from apical papilla (SCAP)
		Bone marrow stromal cells (BMSC)
		Alveolar Bone-Derived MSC (ABMSC)
		Periosteum-Derived MSC (PSC)
		Gingival Stem Cells after Wounds (GMSC)
		MSC Derived from Periapical Cysts (hPCy-MSC)
		Adipose-derived stem cells (ASCs)
		Peripheral blood mesenchymal stem cells (PBMSC)

## ESCs and iPSCs

 ESCs are self-renewing, multipotent cells obtained from embryonic inner cell clusters typically at the blastocyst stage, about 4‒5 days after fertilization.^31^ iPSCs are obtained from adult somatic cells such as skin or blood and are reprogrammed to revert to have the potential to reproduce different types of cells and share comparable properties with ESCs.^32^

 ESCs and iPSCs have tumorigenic characteristics, which is the main problem when applying them for regenerative treatments. The superiority of iPSCs compared to ESCs is in dealing with fewer ethical concerns. In experimental and animal studies, human-derived iPSCs could reproduce all cellular assemblies, including all three primary germ layers. As a resource of iPSCs, cells from dental tissues can be triggered to produce osteogenic cells to regenerate the periodontal bony defects. It has been demonstrated that mouse iPSCs incorporating scaffolds like EMD gel can improve periodontal regeneration, but the use of ESCs is controversial due to ethical concerns regarding the destruction of embryos to obtain them.^31^

## Somatic stem cells

###  DPSCs

 In 2000, Gronthos et al^33^ identified dental pulp stem cells (DPSCs). In vitro, DPSCs can differentiate into odontoblasts, osteoblasts, chondrocytes, and myoblast-resembled cells. DPSCs have the same specifications as bone marrow stromal cells (BMSCs) but have a higher proliferation rate and lower osteogenic capacities. The most common dental pulp stem cell resources are wisdom teeth and first premolars. The dental pulp’s main purpose is to generate primary dentin during tooth development. As the tooth matures, the pulp produces secondary dentin that progressively matures. In response to pathological stimuli, the pulp generates tertiary dentin, a protective mechanism against external threats. The immature stem cells within the dental pulp can transform into odontoblasts, fibroblasts, and other cell types essential for pulp function and regeneration. Various growth factors and odontotropic agents can influence this differentiation process.^34^

###  CDPSCs

 Carious dental pulp stem cells (CDPSCs) are extracted from human teeth with deep caries. Compared to DPSCs, CDPSCs have shown a higher potential for proliferation, mineralization, and expression of osteogenic and dentinogenic genes, attributed to more active angiogenesis activity than normal tissue.^35,36^ Their exact biological characteristics are unclear, but they have shown more colony formation compared with non-carious tissue, which could be attributed to more vascular endothelial growth factor expression in these cells. However, the capabilities of these cells need further investigation.^37^

###  SHEDs

 Stem cells from human exfoliated deciduous teeth (SHEDs) were discovered in 2003. As Miura et al^38^ first described, harvesting SHEDs is a simple, convenient procedure with minimal stress. Enzymatic digestion and tissue transplantation are the two recognized techniques for cultivating SHEDs. The pulp tissues of the deciduous teeth are separated for enzymatic digestion with dispase and collagenase to isolate stem cells. The latter method allows stem cells to proliferate clonally by placing mechanically minced pulps on the tissue culture plates.^39-41^ Based on studies, SHEDs can transform into more cells than DPSCs, such as osteoblast-like, odontoblast-like, and adipocytes.^42^ Based on recent studies, SHEDs originating from deciduous teeth have characteristics similar to the umbilical cord, and they can reproduce more cell groups. The superiority of SHEDs over DPSCs is a higher level of proliferation, but SHEDs cannot regenerate a dentin-pulp-like complex. SHEDs have the advantage of not requiring immune system suppression during transplantation, and their application raises fewer ethical concerns. SHEDs could be obtained from intact primary incisors, canines, and primary molars extracted early for orthodontic treatments.^43^

###  PDLSCs

 Periodontal ligament stem cells (PDLSCs) can be obtained from the perivascular wall of mature PDLs and the middle third of the root surface of extracted permanent dentition. PDLSCs with different origins have different properties. Table 2 shows the types of PDLSCs and their characteristics. Compared to BMSCs, PDLSCs have a higher capacity for the regeneration of adipocytes and osteoblasts. According to studies, the regeneration and transplantation potential of PDLSCs has an inverse relationship with donor age.^44^ PDLSCs have a morphology similar to fibroblasts and can form collectives with high proliferation rates. They can also regenerate collagen filaments, cementum, and Sharpey’s fibers, which are PDL threads that connect to the periosteum of the jawbone and are capable of tissue regeneration.^45^

###  DFSCs

 The tooth germ can be obtained from the third molar of 10‒16-year-old humans. Different parts like the enamel organ and the dental papilla create the tooth germ. Also, the dental follicle is an elastic connective capsule of ectomesenchymal origin encircling the tooth germ during the developing stages, which has an essential effect on tooth eruption. For the first time, dental follicle stem cells (DFSCs) were obtained from animal models in 1992 and the human 3rd molars in 2005. DFSCs can transform into osteoblasts, cementoblasts, PDL cells, and other cell types; therefore, they can be helpful in periodontal regeneration.^46-49^ DFSCs have more extraordinary proliferative ability, and their protein expressions closely resemble those of neural crest cells.^50^ DFSC can regenerate the tooth root, bone, cement, PDL, and dentin pulp-like tissues.^51^

###  SCAPs

**Figure 3:**
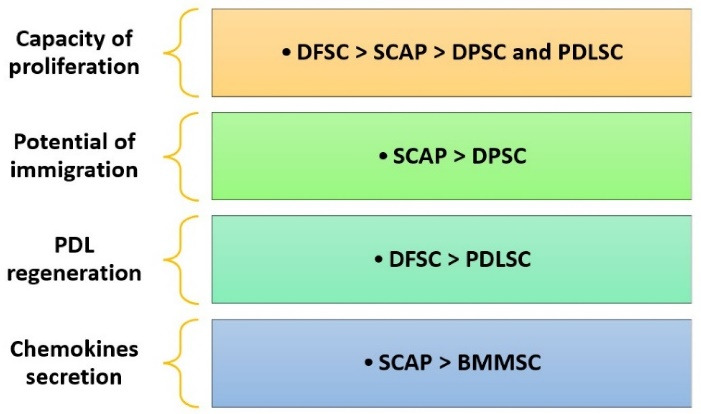
This figure compares the capacities of dental follicle stem cells (DFSC), stem cells from the apical papilla (SCAP), dental pulp stem cells (DPSC), periodontal ligament stem cells (PDLSC), and bone marrow mesenchymal stem cells (BMMSC) in terms of their regenerative potential

 Stem cells from the apical papilla (SCAP) were initially introduced by Sonoyama et al^52^ and obtained from the apical area of a developing permanent tooth. Based on studies, SCAPs can be transformed into cells like osteoblasts, odontoblasts, adipocytes, chondrocytes, and hepatocytes. Figure 3 compares SCAPs with other types of stem cells.^53-56^ Some advantages of SCAPs include their application in the healing and regeneration of pulp in immature teeth and their high survival rate.^57^ Some studies acknowledge that SCAPs are an unrivaled group of postnatal stem cells because of the CD24 markers.^58^ The protein CD24 is highly glycosylated, as demonstrated by various cell types, primarily immunological and central nervous system cells. It appears as a signaling molecule that regulates cell homeostasis, proliferation, and differentiation.^59^ Due to osteoinduction conditions in culture, SCAPs start downregulating their expression of CD24 while increasing the synthesis of alkaline phosphatase.^58^ According to recent research, CD24 is required for SCAPs to undergo optimal adipogenic differentiation; however, it does not affect the osteogenic differentiation of SCAPs. Additionally, CD24 may enhance SCAP adipogenic differentiation by regulating PPAR g2mRNA expression.^59^

###  BMSCs

 Bone marrow is among the primary resources of MSCs.^60^ The capacity of BMSCs to multiply extensively and differentiate into different cell types has been exceptional; nevertheless, their application for periodontal abnormalities has produced mixed results. BMSCs in three-wall intra-bony defects demonstrated limited influence on bone regeneration; however, grade III furcation defects and fenestrations showed more significant responses by increased bone regeneration, indicating that the structural characteristics of the defect are intimately linked to the efficacy of these cells.^61^ According to an experimental study, biphasic scaffold integrating intrafibrillar mineralized collagen (IMC) and concentrated growth factors synergistically enhance the regeneration of periodontal tissues.^62^

###  ABMSCs

 Alveolar bone-derived mesenchymal stem cells (ABMSCs) can be isolated and expanded with a 70% success rate from the human mandibular alveolar bone. These cells can be obtained conveniently from extracted third molar sites or during implant osteotomy procedures. Compared to BMMSC, ABMSC exhibits higher osteogenic differentiation but lower chondrogenic and adipogenic differentiation. Also, it appears that ABMSCs have more potent immunomodulatory qualities than BMSCs.^63-65^

###  PSCs

 For the first time in 1932, Dr. Fell^66^ successfully cultivated the periosteum-derived MSCs (PSCs) and reported that they formed a mineralized matrix with the osteogenic potential of the periosteum. A proper source of PSCs in the oral cavity is the internal layer of the periosteum.^67^ PSCs can be transformed into adipocytes, osteoblasts, and chondrocytes. They can be applied in sinus elevation procedures and reconstruction of large bony defects. PSCs have more potential for creating new bone due to their osteogenic capacity than BMSCs and could be a better choice in regenerating periodontal defects.^66,68-71^

###  GMSCs

 Zhang et al^72^ isolated and described gingival mesenchymal stem cells derived from wounds (GMSCs) in 2009 for the first time. GMSCs are frequently isolated from wound sites because of their high immunoregulatory properties and infection resistance, making them perfect for accelerating tissue regeneration and wound healing.^73^ GMSCs have gained interest for their multipotent nature and ability to transform into diverse cell groups such as osteocytes, odontoblasts, and myocytes. Their immunomodulatory properties and multilineage differentiation capabilities make them suitable for promoting tissue regeneration.^74,75^ The apparent benefit of gingival stem cells is their easy isolation from the gingival tissue following tooth extraction or a biopsy. GMSCs, in conjunction with a hydrogel scaffold, have been applied for the regeneration of maxillary alveolar bony defects.^76^

###  hPCy-MSC

 The MSC derived from periapical cysts (hPCy-MSCs) was obtained from human periapical cysts in 2013. Adipocytes and osteoblasts are among the cell types that can be produced from hPCy-MSCs. These cells are viable for regenerative purposes because of their many advantages, including easy harvest without harming adjacent healthy tissues. They also have proangiogenic and immunomodulatory capabilities. One of their main attributes is being a core part of the bone regeneration process in the periapical area. To regenerate the intended area, they can be added to different scaffolds, such as chitosan and polylactic-co-glycolic acid. However, more studies are needed before they can be used as a routine source of stem cells.^77-79^

###  ASCs

 Gimble and Guilak^80^ introduced adipose-derived stem cells (ADSCs) in the early 2000s. The adipose tissue has 100‒500 times higher concentrations of stem cells than marrow; therefore, it could be a better choice than BMSC. ADSCs have significant benefits due to the abundant adipose tissue and the minimally invasive isolation methods.^81^ According to a recent systematic review, ADSCs have been used in bone tissue engineering for defects associated with dental implants and periodontal defects due to their unique abilities. They have also demonstrated the capability to regenerate pulp tissue in animal studies.^82^ However, more studies are required before they can be used in clinical practice.

###  PBMSCs

 Peripheral blood mesenchymal stem cells (PBMSCs) are promising avenues for periodontal regeneration due to their multipotent differentiation capacity and ease of collection compared to other stem cell sources. These cells can differentiate into osteoblasts, fibroblasts, and other cell types critical for regenerating periodontal tissues. PBMSCs can be harvested through minimally invasive techniques and exhibit immunomodulatory properties, reducing inflammation and promoting tissue repair. The stem cells can be combined with a platelet-rich fibrin matrix as a scaffold in bony defects. Advances in scaffold technologies and biomaterials have further enhanced the delivery and efficacy of PBMSCs in periodontal applications. Their ability to promote angiogenesis and secrete growth factors makes them a vital tool in regenerative periodontal therapies, offering a biologically driven alternative to traditional treatments.^83^

## Preclinical studies

 Animal studies are essential in researching stem cells for periodontal defects because they offer substantial insights that cannot be obtained from in vitro studies (Table 3). Periodontal tissues consist of a complex structure in the oral environment. Animal models are more effective for studying regeneration because they enable biopsy collection for histologic analysis, which provides a direct, detailed view of tissue regeneration. This approach offers more precise insights into cellular behavior and tissue structure than clinical or radiographic assessments. These preclinical studies provide a domain where the interaction of stem cells with these structures can be studied in a living system. They also help detect potential adverse effects, such as immune reactions, unwanted differentiation, or tumor formation. For example, rodent models are advantageous for examining essential biological processes and mechanisms in periodontal regeneration. Although they may not entirely emulate the intricacy of human periodontal disease, they function as pivotal preliminary platforms for evaluating the viability of stem cell therapy. Studies on pigs can also yield helpful information because the periodontium and tooth anatomy of these animals are similar to that of humans.^84^

 The safety and efficacy of a novel injectable material composed of PDLSCs encapsulated in calcium phosphate cement (CPC) paste and biodegradable alginate fibers were evaluated using rat models. Five experimental groups were studied, including CPC alone and CPC combined with varying metformin concentrations. The research assessed the mechanical properties, release, injectability, and osteogenic differentiation of PDLSCs. The alginate fibers degraded within seven days, releasing PDLSCs and significantly enhancing cell proliferation. The findings suggest that the injectable CPC formulation with PDLSCs and 0.1% metformin exhibits exceptional potential for bone regeneration.^85^

 Zhang et al^86^ evaluated the efficacy of using PDLSCs in bony defects in mini pigs. They compared two different scaffolds for carrying the stem cells. IMC was compared with hydroxyapatite (HA) scaffolds. IMC closely replicates the natural organization of bone’s extracellular matrix by incorporating mineralized collagen fibrils at the nanoscale, facilitating cell adhesion, proliferation, and differentiation. The results were evaluated after 12 weeks. The percentage of new bone was about 45% in the IMC and 29% in the HA group, and the nano-structure of the new bone formation was closer to that of natural bone in the IMC group, which proved to be a better scaffold for PDL stem cells.

 In another animal study, bone marrow was collected from the iliac crests of nine mongrel dogs, and the stem cells were cultivated. These cells were integrated with inorganic bovine bone mineral (ABBM) and inserted into surgically created intra-bony periodontal defects. The control group was treated solely with ABBM. Two months after implantation, histological and histometric evaluations were conducted. The results indicated that the test group, which received the BMSCs-loaded ABBM, showed significantly greater novel cementum and PDL regeneration than the control group. These findings suggest that BMSCs combined with ABBM have substantial potential for periodontal regeneration.^87^

 Another study explored the potential of gingival margin-derived stem cells (G-MSCs) for periodontal regeneration combined with a synthetic extracellular matrix. The researchers created periodontal defects in four sites of eight miniature pigs. G-MSCs were isolated from the free gingival margin of the pigs and tested for progenitor cell characteristics. These cells were then expanded and incorporated into the matrix. The study assessed several clinical and radiographic factors, bone and cementum regeneration, junctional epithelium length, and connective tissue attachment at 16 weeks. The results showed that the G-MSCs exhibited characteristics of stem cells. When combined with the matrix, there was significant improvement in clinical outcomes, including bone and cementum regeneration and less gingival recession, compared to the control group. The combination also improved bleeding on probing. The study concluded that G-MSCs have significant potential for periodontal regeneration.^88^

 Iwasaki et al^16^ investigated the PDLSC-amnion’s capacity for regeneration in periodontal defects of a rat model. Amniotic membranes were used to transfer cultivated PDLSCs using photolithography and a surface made of glass coated with polyethylene glycol. PDLSC properties were investigated using experimental differentiation and flow cytometry. Periodontal defects surgically established in maxillary molars were transplanted with PDLSC-amnion. Periodontal regeneration was examined after four weeks using histology and micro-computed tomography. PDLSCs and MSCs shared the expression of cell-surface proteins such as CD90, CD105, CD146, and STRO-1 and the ability to differentiate into osteoblasts. Despite the motion and deformation restored by surgical instruments, the amnion-derived PDLSCs showed one even layer of PDLSCs on their membranes and retained durability. According to these findings, amnion-derived PDLSCs could be used to regenerate periodontal defects.

 PDLSCs can also be used as growth factor carriers to enhance the regenerative process further. Jung et al^89^ analyzed the potential of engineered PDLSCs to express bone morphogenetic protein 2 (BMP2) in mice. These cells formed more dense bone than the control group, showing more osteogenic potential. Khorsand et al^90^ determined how DPSCs affected the reconstruction of experimentally created periodontal lesions in a canine model. Ten dogs of mixed breeds had their first inferior premolars surgically altered to have proximal three-walled periodontal defects on both sides caused by ligature-induced periodontitis. The maxillary premolars of the same dogs were used to harvest DPSCs. Almost one month later, the experimental case was established, and a self-derived third-passage mixture of DPSCs with Bio-Oss was inserted into one side (test group). In contrast, Bio-Oss alone was introduced on the opposite side (control group). After eight weeks, regeneration was observed histologically and morphometrically, including bone, PDL, and cementum.

 In a canine model, Tobita et al^91^ evaluated whether combining ASCs with platelet-rich plasma (PRP) could enhance periodontal regeneration. PRP and self-derived ASCs were applied to sites with periodontal defects, while control sites received only PRP or no treatment. Histological, immunohistochemical, and radiographic analyses were performed one and two months after treatment. The regenerated cementum and bone in the defect areas were measured. The results showed that regeneration was significantly greater two months after treatment compared to one month, with noticeable increases in radiopacity in the defect regions by two months. The group treated with ASCs and PRP demonstrated proper regeneration at two months. The study concluded that self-derived ASCs and PRP effectively promoted periodontal tissue regeneration, helping to create the necessary framework for this complex tissue.

 In Tsumanuma and colleagues’ study,^92^ three types of stem cells, PDLSCs, ASCs, and BMSCs, were tested for periodontal regeneration using cell sheet transplantation. One-wall intra-bony defects were created in dogs to compare the effects of each cell source. Three layers of autologous cells from each group were grafted onto the exposed root surfaces and supported with woven polyglycolic acid. Collagen combined with β-tricalcium phosphate (β-TCP) was used to treat the defects. After two months, the PDLSC group showed the most significant periodontal regeneration, with noticeable new cementum formation and well-aligned PDL fibers. These results suggest that PDLSC sheets combined with a β-TCP/collagen matrix have a strong potential for periodontal regeneration.

 Duan et al^93^ explored the opportunities and advantages of using EMDs and iPSCs to regenerate periodontal tissue. They initially assessed the impact of EMD gel on iPSCs in vitro, followed by a tissue engineering technique for periodontal regeneration. The study involved three experimental groups: one with a silk scaffold alone, another with EMD, and a third group combining EMD with iPSCs. After 24 days, the group treated with iPSCs showed significantly greater periodontal tissue regeneration. The findings demonstrated EMD’s role in guiding mesenchymal progenitors toward osteogenic differentiation and underscored the synergistic potential of combining iPSCs with EMD to enhance periodontal regeneration.

 Animal studies have consistently shown that stem cell-based treatments can promote the regeneration of alveolar bone and cementum, essential components of the periodontium. However, while short-term results in animal studies are promising, long-term studies are needed to ensure the sustained regeneration of periodontal tissues and prevent relapse or failure of the regenerated tissues.

## Clinical studies

 In different clinical trials, MSCs of various origins, including dental pulp, PDL, gingiva, bone marrow, and peripheral blood, have been applied to reconstruct the periodontal defects in periodontitis patients (Table 4). These stem cells can be combined with other growth factors, such as platelet fibrin matrix, to increase the chance of regeneration in periodontal defects. Sreeparvathy et al^83^ combined stem cells from the blood with this matrix, manufacturing a “supercell” material for defects with pocket depths of ≥ 6 mm. They designed a randomized clinical trial and followed their patients up to 6 months. Adding the stem cells to the fibrin matrix increased its efficacy, which is evident in clinical and radiographic analyses, and no adverse reactions were observed in any of the patients.

**Table 2:** Different types of periodontal ligament stem cells (PDLSC) and their characteristics

**Type of PDLSC**	**Characteristics**
Root surface derived PDLSC (r-PDLSC)	Lower capacity of adipogenic and osteogenic regeneration compared to a-PDLSC
Alveolar bone derived PDLSC (a-PDLSC)	Higher capacity of adipogenic and osteogenic regeneration compared to r-PDLSC
Deciduous teeth derived PDLSC (d-PDLSC)	Higher proliferation rate and adipogenic and osteogenic capacity compared to p-PDLSC
Permanent teeth derived PDLSC (p-PDLSC)	Lower proliferation rate and adipogenic and osteogenic capacity compared to d-PDLSC
Inflammatory PDL stem cell (i-PDLSC)	Higher proliferation and faster migration but lower cementogenic and osteogenic capacity compared to h-PDLSC
Healthy PDL stem cell (h-PDLSC)	Lower proliferation and slower migration but higher cementogenic and osteogenic capacity compared to h-PDLSC

**Table 3:** Preclinical animal studies on stem cell therapy for periodontal regeneration

**Author**	**Year**	**Cell type**	**Animal model**	**Case group**	**Control group**	**Follow-up period**	**Outcome**
Sun et al^85^	2023	PDLSC	Rats	CPC + PDLSC CPC + PDLSC + 0.1% metformin CPC + PDLSC + 0.2% metformin CPC + PDLSC + 0.4% metformin	CPC scaffold	1 week	CPC + PDLSC + 0.1% metformin group exhibited the highest osteogenic differentiation
Zhang et al^86^	2017	PDLSC	Mini Pig	IMC	HA	12 weeks	IMC is better than HA for carrying PDLSC
Paknejad et al^87^	2015	BMSC	Dog	ABBM + BMSC	ABBM	8 weeks	Combining BMSCs with ABBM improves bone regeneration
Fawzy et al^88^	2015	GMSC	Mini Pig	GMSC	Access Flap	16 weeks	GMSC improves clinical parameters
Iwasaki et al^16^	2014	PDLSC	Rat	PDLSC + Amniotic membrane	amniotic membrane only	4 weeks	PDLSC + amnion shows better periodontal regeneration
Jung et al^89^	2014	PDLSC	Mouse	PDLSC + BMP2	Scaffold	3 to 7 days	Applying the PDLSC as cellular carriers for BMP2 shows promising results
Khorsand et al^90^	2013	DPSC	Dog	DPSC + Bio-Oss	Bio-Oss	4 to 8 weeks	DPSCs enhance periodontal regeneration
Tobita et al^91^	2013	ASC	Dog	PRP + ASC	PRP only	4 to 8 weeks	combination of ASC and PRP improves regeneration
Tsumanuma et al^92^	2011	PDLSC BMSC APC	Dog	Stem cells + β-TCP/collagen	β-TCP/ collagen	8 weeks	PDLSCs are the most appropriate for periodontal regeneration
Duan et al^93^	2011	iPSC	Mouse	silk scaffold + EMD + iPS cells	silk scaffold only	12 to 24 days	Combining iPSC with EMD significantly improves the healing of mouse periodontal defects by facilitating the formation of cementum, alveolar bone, and a functional PDL

PDLSC (periodontal ligament stem cells), CPC (calcium-phosphate cement), IMC (intrafibrillar mineralized collagen), HA (hydroxyapatite), BMSC (bone marrow stem cells), ABBM (anorganic bovine bone mineral), GMSC (gingival mesenchymal stem cells), BMP2 (bone morphogenetic protein 2), DPSC (dental pulp stem cells), ASC (adipose-derived stem cells), PRP (platelet-rich plasma), APC (alveolar periosteal cells ), β-TCP (beta-tricalcium phosphate), iPSC (induced pluripotent stem cells), EMD (enamel matrix derivative), PDL (periodontal ligament).

**Table 4:** Clinical studies on stem cell therapy for periodontal regeneration

**Author**	**Year**	**Type of Study**	**Cell type**	**Case group**	**Control group**	**Follow-up period**	**Outcomes**
Sreeparvathy et al^83^	2024	Randomized clinical trial with a split-mouth design	PBMSC	PRFM + PBMSCs	PRFM alone	6 months	More pocket reduction and CAL gain
Apatzidou et al^94^	2021	A Phase I/II double-blind randomized controlled clinical trial	BMSC from alveolar bone	BMMS on collagen scaffolds enriched with fibrin/platelet lysate	No stem cells	12 months	No adverse healing, 3.0 mm attachment gain, 3.7 mm pocket reduction, 0.7 mm recession
Hernández-Monjaraz et al^95^	2020	Quasi-experimental study	DPSC	DPSC	No stem cells	6 months	Bone regeneration, reduced oxidative stress, and reduced inflammation
Abdal-Wahab et al^96^	2020	Randomized clinical and biochemical trial	GMSC	GMSC + β-TCP + collagen membrane	β-TCP + collagen membrane	6 months	More pocket depth, CAL gain, radiographic bone gain, and higher PDGF-BB
Sánchez et al^97^	2020	Quasi-randomized controlled pilot clinical trial	PDLSC	Xenogeneic bone substitute with PDLSC	Xenogeneic bone substitute	12 months	Safe but no added benefit compared to the control group
Ferrarotti et al^98^	2018	Randomized controlled clinical trial	DPSC	DPSC on a collagen sponge	collagen sponge	12 months	More pocket reduction, CAL gain, and bone fill
Iwata et al^99^	2018	Single-arm and single-institute clinical study	PDLSC	PDLSC + β-TCP	Not present	55 months	PDLSC + β-TCP is safe and effective
Shalini et al^100^	2018	Randomized controlled clinical trial	PDLSC	PDLSC	No stem cells	12 months	More pocket reduction, CAL gain, and increase in defect density
Baba et al^101^	2016	Phase I/II clinical trial	BMSC from the iliac bone	BMSC + composite scaffold + PRP	No stem cells	36 months	Safe and effective with 4.7 mm Linear bone growth
Chen et al^102^	2016	Randomized clinical trial	PDLSC	PDLSC + GTR + Bio-Oss	GTR + Bio-Oss	12 months	Safe but no added benefit compared to the control group

PBMSC (peripheral blood mesenchymal stem cells), PRFM (platelet-rich fibrin matrix), CAL (clinical attachment level), BMSC (bone marrow stem cells), DPMSC (dental pulp stem cells), GMSC (gingival mesenchymal stem cells), β-TCP (beta-tricalcium phosphate), PDGF-BB (platelet-derived growth factor-BB), PDLSC (periodontal ligament stem cells), DPSC (dental pulp stem cells), GMSC (gingival mesenchymal stem cells), BMSC (bone marrow stem cells), PRP (platelet-rich plasma), GTR (guided tissue regeneration).

 Apatzidou et al^94^ evaluated the effectiveness and safety of three treatments for intrabody defects in a cohort of 27 patients. Participants were randomly divided into three categories. Autologous BMMSCs were given to group A. These cells were enhanced with fibrin/platelet lysate and then incorporated into collagen substrates. The scaffold without the stem cells was used in group B. An access flap without any scaffolds or stem cells was used for the third group. No adverse healing events were reported in the first year. Group A showed better results than group B, illustrating the role of stem cells in stimulating bone regeneration.

 A quasi-experimental study assessed the impact of dental pulp mesenchymal stem cells (DPMSCs) on periodontal disease in a cohort of 22 middle-aged adults. The stem cells were loaded on collagen scaffolds in the test group, and the scaffolds alone were used for the control group without the stem cells. Clinical and radiographic re-evaluations were carried out after six months. The test group demonstrated significant increases in bone mineral density and superoxide dismutase levels alongside reduced IL-1β levels, suggesting that DPMSC therapy enhanced bone regeneration and reduced inflammatory markers and oxidative stress in periodontitis patients.^95^

 Abdal-Wahab et al^96^ assessed the effects of GMSCs in intra-bony periodontal defects using clinical and biochemical methods. GMSCs were added to β-TCP and a collagen barrier in the test group. In the control group, the stem cells were not used. The test group showed a more significant decrease in pocket depth and increased clinical attachment level (CAL) and bone formation. Additionally, greater levels of PDGF-BB were seen in the GMSC group shortly after therapy, demonstrating its potential usefulness in improving growth factors in the defect.

 Although stem cells are a safe choice for periodontal regeneration, some studies have not reported an added benefit compared to bone grafts alone. For instance, xenogeneic bone substitutes have been beneficial in periodontal bony defects. However, adding PDLSCs did not lead to significantly more regeneration when added to the bone graft after 12 months in 1‒2-wall intra-bony defects.^97^

 A randomized clinical trial was conducted by Ferrarotti et al,^98^ including 29 patients with chronic periodontitis. After removing the tooth scheduled for extraction, it was cleaned for 60 seconds in 0.2% chlorhexidine. After that, it was given to the study coordinator in charge of isolating DPSCs. The coordinator used a Gracey curette to remove the pulp tissue and cut the tooth along the cementoenamel junction to reveal the pulp chamber. Patients in the test group were treated with DPSCs on a collagen sponge, and the control group received the collagen sponge alone. One year later, the test group exhibited more pocket reduction, CAL gain, and regeneration of bone.

 Another clinical study analyzed the reliability and potency of autologous PDL stem cell sheets in patients with periodontal defects who underwent tooth extractions. The extracted tooth was used for harvesting PDLSCs, which were transplanted to the defect using standard flap surgeries and then filled with beta-tricalcium phosphate granules. The results demonstrated improvements in clinical and radiographic parameters up to 55 months after the surgery. This approach was reportedly secure and efficacious for treating defects in severe periodontitis.^99^

 Shalini and Vandana^100^ assessed the impact of direct transplantation of PDLSC niches on intra-bony defects. The study included 28 patients in two groups: 14 received open flap debridement (OFD) with PDLSC niche transplantation, and the other 14 received OFD alone. Both groups exhibited significant clinical improvements, but the cell therapy group showed higher enhancement in defect density.

 A novel regenerative treatment employing self-derived MSCs with a 3-D woven-fabric compound framework and PRP evaluated the participants with periodontal diseases. Ten patients underwent surgical implantation of MSCs that were extracted from each subject’s iliac bone marrow by aspirating it one month before the periodontal surgery. The patients were monitored for 36 months. In this study, CAL, pocket depth, and linear bone growth (LBG) were evaluated, and based on the results, an average LBG of 4.7 mm was obtained in three years. This study concluded that MSC therapy is a harmless and successful intervention for periodontal defects.^101^

 Another clinical study investigated the efficacy of PDLSCs combined with bovine bone mineral components. Thirty cases of periodontal disease were randomly assigned to either the cell group, which received PDLSC plates with Bio-Oss and GTR, or the control group, which received GTR and Bio-Oss without stem cells. There were no statistically significant differences between the two groups, but both showed notable increases in alveolar bone levels. There were no documented safety issues with the experimental PDLSCs.^102^

 All the studies mentioned above proved stem cells safe; however, some studies did not observe an added benefit for using stem cells compared to routine regenerative techniques.

## Discussion

 Periodontal regeneration techniques include GTR, growth factors like EMD, and stem cell therapy, each with distinct advantages and limitations. GTR uses barrier membranes to guide the regeneration of periodontal tissues, offering proven success in treating intra-bony and furcation defects. Still, it requires precise surgical technique, has a risk of infection from membrane exposure, and usually results in long junctional epithelium instead of true regeneration. Growth factors like EMD stimulate natural healing processes and are minimally invasive, making them suitable for shallow or single defects. However, their efficacy depends on patient factors, and they are expensive. Stem cell therapy, a cutting-edge approach, holds the promise of true tissue regeneration, particularly for extensive defects. Still, it remains experimental, mainly costly, and technically demanding, with ethical and regulatory challenges. The choice of technique depends on the defect type, patient-specific factors like systemic health and smoking status, and clinician expertise, with combination approaches often yielding the best results.

 True regeneration can only be assessed through histologic evaluations, although most relevant studies have predominantly evaluated radiographic and clinical parameters. Animal studies using stem cells have reported true periodontal regeneration, which was assessed histologically. In a study by Hu et al,^103^ histological evaluations verified that using DPSCs yielded actual periodontal bone regeneration in swine, with increased bone percentages and new cementum-like layers. Additionally, the test group showed positive expression of human β-globin and new Sharpey fibers. In another study, histological analyses showed that the bone marrow stem cells significantly increased periodontal tissue regeneration compared to the control groups.^104^ MSCs are commonly employed for periodontal regeneration because they can multi-differentiate. Extraoral stem cells such as BMSC and ASC and intraoral stem cells like PDLSC, DPSC, SCAP, DFSC, and SHED are receiving much attention in tissue engineering.^105^

 Direct and indirect comparisons have demonstrated that DPSCs are more effective for bone regeneration, while ADSCs promote cementum regeneration.^106^ Other studies have examined the efficacy of cell therapies in more complex experimental models, such as Cl III furcation or supra-alveolar defects. However, these investigations have yielded inconclusive results, failing to demonstrate a clear advantage of cell therapy. In such scenarios, choosing the proper carrier and precise management of the soft tissues are essential to prevent the regenerative technology from being exposed.^107^

 Exosomes are extracellular vesicles containing certain substances with biological activity, such as proteins and nucleic acids, which can induce regeneration. Various cell types, such as stem cells, dendritic cells, and epithelium cells, secrete exosomes. They are present in saliva in the oral cavity. Stem cell-based exosomes are a novel therapeutic approach because of their capability for regeneration. The exosomes secreted from oral source stem cells, such as PDLSC-exosomes and DFSCs, are beneficial in ameliorating the inflammation of periodontal tissues and improving periodontal regeneration and repair. The SHED-exosomes improve periodontal regeneration better than periodontal membrane cell-derived exosomes.^108,109^ Exosomes derived from SCAP exhibit significant promise for oral tissue regeneration. These exosomes may be a proper interventional method for dentin‒pulp complex regeneration. Furthermore, angiogenesis and soft tissue regeneration in a mouse model were markedly improved by the injection of SCAP-derived exosomes into the palatal gingival area.^108,110^ PDLSC exosome activity promotes proliferation, angiogenesis, and osteogenesis while controlling inflammatory responses, all of which help maintain periodontal homeostasis.^111^

 Recently, research focused on MSC-derived small extracellular vesicles (sEV) as a potential substitute for stem cell therapy.^112^ sEVs offer advantages over MSC-based therapies, which face significant logistical challenges in manufacturing, handling, delivering, and storing cells for transplantation. They are ready to use and can be easily reformulated for various administration routes. Moreover, sEVs are easy to store and maintain biological activity during long-term storage, reducing the cost and time required for stem cell expansion from patients.^113-117^ The accessibility of stem cells is also challenging. Significant amounts of stem cells are required for treating periodontal defects, ranging from 5 × 10^4^ to 2 × 10^8^ cells per defect. This quantity is often challenging to procure from a single patient. While it is possible to cultivate stem cells in vitro, their capacity for self-renewal and proliferation typically diminishes with each passage.^118^ Another drawback of stem cell therapy is its lack of long-term evidence of its success. More controlled studies are needed before stem cells can be used in daily practice.^119,120^

## Conclusion

 This study reviewed the potential of stem cells in regenerating periodontal bony defects, highlighting their diverse capabilities and limitations. PDLSCs demonstrated superior ability in producing osteoblasts, making them highly effective for regeneration. DFSCs showed versatility by regenerating multiple periodontal structures, including the ligament, cementum, and bone. ADSCs offer advantages due to their abundance and minimally invasive collection methods, while BMSCs were less successful in addressing intra-bony lesions. Despite their promise, challenges remain, including potential tumorigenic risks, ethical considerations, and variability in efficacy across different defect types. Future research should focus on optimizing the application of stem cells to enhance regenerative outcomes while addressing these challenges.

## Competing Interests

 The authors declare that they have no conflicts of interest.

## Data Availability Statement

 All data are included in this published article.

## Ethical Approval

 Not applicable.
